# The New Medical School at Chicago

**Published:** 1859-12

**Authors:** 


*The New Medical School at Chicago.*—From Prof. N. S. Davis's journal we learn that the new school organized by himself and others (the Medical Department of Lind University,) opened with a class of eighteen, which has ultimately increased to twenty-six—there being fourteen in the junior and twelve in the senior department. Being the practical advocates of improvements in medical teaching in this country, we cannot be understood as having the least desire to chill the ardor of those engaged in the same honorable pursuit; and we hope our motive may be thoroughly appreciated when we express our fears that the innovation of our Chicago friends have been too thorough.—A good old maxim is, *festina lente.* Step by step we can revolutionize the system of teaching, but it cannot be done in a day.

We fear the faculty of Lind University will waste their valuable strength in too sudden and radical changes. We hope not.—Their motives are noble, and they deserve the fullest reward.—But they must have the substantial aid of larger classes, and that speedily. We speak from experience. We have succeeded, and know what is necessary to success.—*N. O. Medical News and Gazette.*

